# Return on investment of public health interventions: a systematic review

**DOI:** 10.1136/jech-2016-208141

**Published:** 2017-03-29

**Authors:** Rebecca Masters, Elspeth Anwar, Brendan Collins, Richard Cookson, Simon Capewell

**Affiliations:** 1 North Wales Local Public Health Team, Public Health Wales, Mold, Flintshire, UK; 2 Department of Public Health and Policy, University of Liverpool, UK; 3 Department of Public Health, Halton Borough Council, Cheshire, UK; 4 Department of Public Health, Wirral Metropolitan Borough Council, Merseyside, UK; 5 Centre for Health Economics, University of York, UK

**Keywords:** PUBLIC HEALTH, ECONOMICS, Health inequalities, PUBLIC HEALTH POLICY, Economic evaluation

## Abstract

**Background:**

Public sector austerity measures in many high-income countries mean that public health budgets are reducing year on year. To help inform the potential impact of these proposed disinvestments in public health, we set out to determine the return on investment (ROI) from a range of existing public health interventions.

**Methods:**

We conducted systematic searches on all relevant databases (including MEDLINE; EMBASE; CINAHL; AMED; PubMed, Cochrane and Scopus) to identify studies that calculated a ROI or cost-benefit ratio (CBR) for public health interventions in high-income countries.

**Results:**

We identified 2957 titles, and included 52 studies. The median ROI for public health interventions was 14.3 to 1, and median CBR was 8.3. The median ROI for all 29 local public health interventions was 4.1 to 1, and median CBR was 10.3. Even larger benefits were reported in 28 studies analysing nationwide public health interventions; the median ROI was 27.2, and median CBR was 17.5.

**Conclusions:**

This systematic review suggests that local and national public health interventions are highly cost-saving. Cuts to public health budgets in high income countries therefore represent a false economy, and are likely to generate billions of pounds of additional costs to health services and the wider economy.

## Introduction

Benjamin Franklin once famously stated that “an ounce of prevention is worth a pound of cure”. Long-term pressures on public sector costs due to demographic and technological changes and cost inflation in the caring professions have intensified following the 2008 global financial crisis. Public health is often considered a politically soft target for budget cuts, as recently demonstrated by major budget reductions in the UK.^1–3^

The benefits of population-level public health expenditure—unlike those of personal healthcare and social care expenditure—tend to be long term, mostly accruing after the current politicians and policymakers have moved on. Though large and certain at the population level, benefits are also seen as small and uncertain for individual voters. It is therefore important to take a hard look at the cost-effective evidence, and move towards more rational decision-making in this politically charged area.

Return on investment (ROI) and cost-benefit ratio (CBR) are two forms of economic evaluation that value the financial return, or benefits, of an intervention against the total costs of its delivery. The CBR is the benefit divided by the cost, and the ROI is the benefit minus the cost expressed as a proportion of the cost, that is, the CBR−1. To help inform the discussion of proposed cuts to public health budgets, we set out to determine the ROI and opportunity cost for a range of public health interventions at the local and national levels. The theory underpinning this review is that, because political backing for public health intervention is often lacking, many interventions with a high ROI are not funded. This is because public health interventions are often opposed by powerful commercial interests, and the health gains for individuals are often perceived as too small to sway their voting intentions, despite adding up to large gains at the population level.^4^

## Methods

We conducted a systematic review to examine the ROI of public health interventions delivered in high-income countries with universal healthcare. These included the UK, Western Europe, the USA, Canada, Japan, Australia and New Zealand.

### Search strategy

The authors used Acheson's definition of public health when considering our search strategy: “The science and art of promoting and protecting health and well-being, preventing ill-health and prolonging life through the organised efforts of society”.^5^ This definition is purposefully broad and the authors felt that it would incorporate the various fields of public health. We searched the PubMed, MEDLINE, Scopus, CINAHL, Cochrane, PsycInfo and AMED databases using the following search terms: ‘public health’ (all fields) AND ‘return on investment’ OR ‘cost benefit analysis’ (title or abstract). We also hand searched the references of the included analyses to identify any further studies. A grey literature search was completed using Google, yielding three additional results. Limits were set to publications in the English language, and to interventions targeted at humans (where applicable). Studies with poor generalisability to the UK were excluded, including a number from the USA that may poorly reflect UK healthcare systems, structure and demographics.

### Study selection and inclusion criteria

We included studies of any design that reported a ROI of public health interventions delivered in industrialised countries providing universal healthcare.

### Selection of articles and extraction of data

One investigator (RM) performed the initial screening of the titles. A second reviewer (EA) independently reviewed the titles and potentially relevant abstracts. The results were cross-referenced and any disagreements were discussed with a third reviewer (BC).

One investigator (RM) led the data extraction and quality assessment, which was then independently duplicated by EA. A third reviewer (BC) adjudicated on any disagreements regarding result details or quality assessment. RM contacted authors for additional data in three cases, with two responses.

### Assessment of methodological quality in included studies

The methodological quality of each included study was assessed independently by two reviewers (RM and EA) using the National Institute for Health and Care Excellence (NICE) quality appraisal checklist for economic evaluations to assess the quality and external validity of each study.^6^ Disagreements in methodological quality assessments for all the included studies were resolved by consensus or by recourse to a third member of the review team (BC).

## Results

We identified 2957 potentially relevant titles, after excluding 2559 duplicates. A further 2816 papers were excluded following title or abstract review. We finally included 52 relevant titles published over four decades (see online supplementary figure S1).

10.1136/jech-2016-208141.supp1 supplementary data

Results were stratified by public health specialty (table 1), and by interventions at a local level (table 2) or national level (table 3). Results were reported in five different currencies, as detailed in tables 2 and 3.

**Table 1:** ROI of public health programmes overall, and stratified by level and specialism

	Median ROI	ROI range	Number of ROI studies	Median CBR	CBR range	Number of CBR studies
Overall	**14.3**	–21.3 to 221	34	**8.3**	0.7 to 29.4	23
Local level	**4.1**	0.9 to 19.3	18	**10.3**	0.9 to 23.6	11
National level	**27.2**	–21.3 to 221	17	**17**	1.2 to 167.	10
Specialism						
Health protection	**34.2**	0.7 to 221	8	**41.8**	1.1 to 167	10
Legislation	**46.5**	38 to 55	2	**5.8**	3 to 8.6	2
Health promotion	**2.2**	0.7 to 6.2	12	**14.4**	2 to 29.4	3
Healthcare public health	**5.1**	1.1 to 19.3	6	None reported	None reported	None reported
Wider determinants	**5.6**	1.1 to 10.8	6	**7.1**	0.7 to 23.6	6

CBR, cost-benefit ratio; ROI, return on investment.

**Table 2:** Return on investment of local public health programmes: specific studies

Reference	Intervention	Population	Benefit-cost ratio	Return on investment	Cost perspective	Discount rate	Time horizon	Study quality
Andresen *et al* 31	Supervised injection facilities	IDU population of Vancouver, Canada	5.12		Medical and societal	3%	Lifetime	++
Arrieta *et al* 18	Home blood pressure monitoring for hypertension diagnosis and treatment	16 375 participants, USA		$7.50–$19.35	Insurer	3%	10 years	++
Baker *et al* 44	Workplace obesity management	890 employees, USA		$1–$1.17	Medical	None	1 year	–
Beard *et al* 41	Community-based falls prevention	2000 cases and 1600 matched controls, Australia	20.6		Medical and societal	8%	18 months	++
Collins^42^	Smoking cessation	Population of Wirral, UK		£1.77	Medical	3.5%	20 years	++
Dopp *et al* 57	Multisystematic therapy with serious juvenile offenders and their siblings	305 participants, USA	5.04		Medical and societal	3%	25 years	++
Goetzel *et al* 45	Workplace health risk management programme for small businesses	2458 employees, USA		$2.03	Medical and productivity	No	1 year	–
Guo *et al* 59	Improved walking and cycling infrastructure	4674 participants, USA	1.87		Medical	3%	10 years	++
Kleitz *et al* 21	Multisystematic therapy with serious juvenile offenders	176 participants, USA	9.51–23.59		Medical and societal	3%	13.7 years	++
Kuehl *et al* 46	Workplace health promotion for fire fighters	1369 fire fighters, USA		$4.61	Medical and insurer	None	7 years	–
Long *et al* 47	Health promotion programme for hospital staff	4402 hospital staff, USA		$2.87	Employer	None	1–4 years	+
Moore *et al* 52	Medication management for high-risk groups	4500 health plan participants, USA		$2	Insurer	None	1 year	–
Medivil *et al* 60	Speed cameras in urban settings	Barcelona, Spain		€6.80	Medical and societal	3%	2 years	++
Nelson *et al* 39	Water fluoridation	Population of Houston, Texas		$1.51	Societal	10%	10 years	++
Nyman *et al* 22	Workplace health promotion	1757 cases and 3619 matched controls, employer, USA		$0.87		No	2 years	+
Ozminkowski *et al* 51	Workplace health management	25 931 Citibank employees		$4.61	Insurer	4%	3.2 years	++
Peters *et al* 9	20 mph zones in London	Population of London, UK	0.66–2.19		Societal	3.5%	10 years	++
Reynolds *et al* 20	Intensive early education programme for socioeconomically deprived families (preschool programme)	1539 participants, USA		$10.83	Medical and societal	3%	20 years	++
Reynolds *et al* 20	Intensive early education programme for socioeconomically deprived families (school age programme)	850 participants, USA		$3.97	Medical and societal	3%	20 years	++
Reynolds *et al* 20	Intensive early education programme for socioeconomically deprived families (extended intervention)	553 participants, USA		$8.24	Medical and societal	3%	20 years	++
Richard *et al* 43	Tobacco cessation	805 Medicaid insured tobacco users, USA		$2–$2.25	Insurer	None	1.3 years	–
Rundell *et al* 53	Therapeutic services for alcoholism	3034 Oklahoma alcohol service users, USA		$1.98	Medical and legal	4%	10 and 22 years	++
Schwartz *et al* 48	Wellness and disease prevention programme	57 940 health insurance clients, USA		$2.02	Insurer	None	8 years	–
Schweinhart *et al* 56	Preschool education programme for socioeconomically deprived children	123 preschool children, USA	7.16		Medical and societal	3%	40 years	–
Steinbach *et al* 19	20 mph zones in London	Population of London, UK		£1.12	Medical	None	10 years	–
Spence *et al* 54	Outpatient pharmacy services for medication adherence	2957 matched cases and controls, USA		$5.97	Medical and productivity	None	1 year	–
Van Vonno *et al* 17	Heart failure disease management	1360 matched cases and controls, USA		$1.15	Insurer	None	1 year	–
Wang *et al* 61	Bike and pedestrian trails	225 351 individual uses of bike and pedestrian trails over a 1 year period, USA		$2.94	Public health	None	10 years	–
Windsor *et al* 49	Antinatal stop smoking services	994 pregnant smokers in Alabama, USA	6.72–17.18		Medical	None	5 years	–

**Table 3:** Return on investment of national public health programmes: specific studies

Reference	Intervention	Population	Benefit-cost ratio	Return on investment	Cost perspective	Discount rate	Time horizon	Study quality
Abelson *et al* 10	Hib vaccination	Australia	1.06		Medical	5%	15 years	++
Abelson *et al* 10	HIV/AIDS prevention	Australia	4		Medical	5%	25 years	++
Abelson *et al* 10	Measles vaccination	Australia	167		Medical	5%	33 years	++
Abelson *et al* 10	Programmes to reduce rates of coronary heart disease	Australia	11		Medical	5%	40 years	++
Abelson *et al* 10	Programmes to reduce tobacco consumption	Australia	2		Medical	5%	40 years	++
Abelson *et al* 10	Road safety campaigns	Australia	3		Medical	5%	40 years	++
Boccalini *et al* 32	Universal hepatitis B vaccination	Italy		€2.78	Medical and societal	3%	20 years	++
Bonin *et al* 58	Parenting programmes for the prevention of persistent conduct disorders	England	7.89		Medical and societal	3.5%	35 years	++
Drummond^11^	Needle exchange	Australia	1.2		Public health	5%	Lifetime	++
Evans-Lacko *et al* 12	Antistigma social marketing campaign	England		£0.7 to £1.90	Unclear	None	1 year	-
Garpenholt *et al* 38	Hib vaccination	Sweden	1.59		Societal	5%	20 years	++
Gortmaker *et al* 15	Sugar sweetened beverage tax	USA		$55	Medical	3%	10 years	++
Gortmaker *et al* 15	Eliminating tax subsidy of nutritionally poor food advertising to children	USA		$38	Medical	3%	10 years	++
Gould^8^	Lead paint control	USA		$17 to $221	Medical and societal	None	Unclear	**−**
Holtgrave *et al* 34	HIV counselling, testing, referral and partner notification services	USA	20.09		Societal	6%	Lifetime	++
Hutchinson *et al* 35	Expanded HIV testing	USA		$1.46 to $2.01	Health sector	3%	Lifetime	++
Kwon *et al* 36	Needle exchange	Australia		$A1.3 to $A5.5	Health sector	3%	Lifetime	++
Lokkerbol *et al* 55	Telemedicine for depression	The Netherlands		€1.45 to €1.76	Medical	1.5% costs, 4% effects	5 years	++
McGuire *et al* 14	Family planning services	UK	11.09 to 29.39		Medical	6%	Lifetime	++
Miller *et al* 16	Booster seats for 4–7 years olds	USA	8.6		Medical	3%	3 years	++
Nguyen *et al* 37	Needle exchange	USA		$3.48	Medical	None	1 year	+
Nichol *et al* 7	Influenza vaccination of healthy workers	USA		**−**$21.27 to +$174.32	Societal	3%	1 year	+
Romano *et al* 40	Folic acid fortification of grain	USA	4.3 to 6.1		Human capital	4%	Lifetime	++
Trust for America^13^	Primary and secondary prevention programmes	USA		$6.2	Medical	0%	10–20 years	+
Wang *et al* 50	Universal school nursing services	USA		$2.20	Societal	None	1 year	+
White *et al* 28	MMR vaccination	USA	14		Medical	10%	Lifetime	++
Ding *et al* 30	Hospital-based postpartum influenza vaccination	USA		$1.7	Medical and societal	3%	1 year	++
Zhou *et al* 38	Hib vaccination	USA	5.4		Medical and societal	3%	Lifetime	++

Hib, haemophilus influenzae type b; MMR, measles, mumps and rubella.

The median ROI for all public health interventions was 14.3, and the median CBR was 8.3.

The reported ROI and CBRs ranged widely. The ROIs ranged from –21.27 (influenza vaccination of healthy workers^7^) to 221 (lead paint control^8^). The CBRs reported ranged from 0.66 (20 mph zones in low-impact areas^9^) to 167 (single measles vaccinations^10^). Studies reporting ROIs at the extreme end of the spectrum tended to be of poorer quality. Studies reporting a CBR tended to be higher quality.

### ROI of public health programmes stratified by specialism

**Figure 1:**
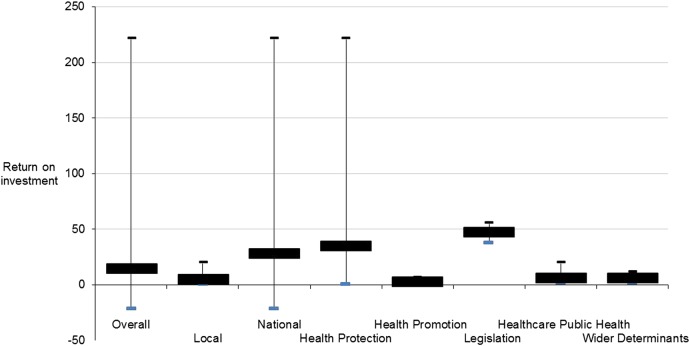
Median, maximum and minimum return on investment values stratified by specialism.

**Figure 2:**
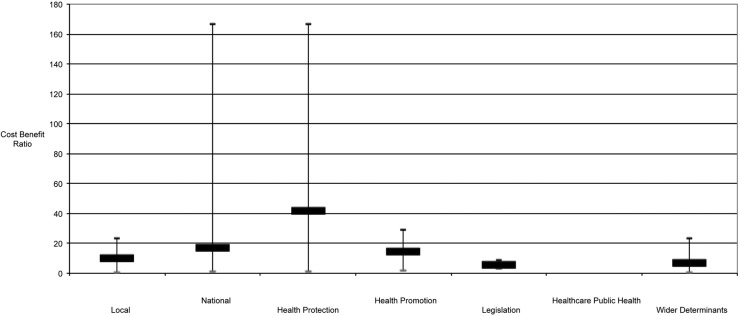
Median, maximum and minimum cost:benefit ratios stratified by specialism.

Analysis by specialism revealed that health protection and legislative interventions generally yielded high returns on investment, often being delivered on a national basis and only requiring a one-off intervention (such as a vaccination or a new tax). In contrast, interventions for healthcare public health, health promotion or wider determinants typically had lower returns, being often more complex, resource intensive and sustained. Figure 1 provides overviews of the median, maximum and minimum ROI by specialism, and figure 2 provides an overview of the median, maximum and minimum and CBR values stratified by specialism.

#### Health protection interventions

Eighteen studies reported a large ROI in relation to health protection. The ROI median was 34.2, and ranged from −21.3^7^ to 221^8^, and the CBR median was 41.8 (range from 1.2^11^ to 167^10^).

#### Health promotion interventions

Fifteen health promotion interventions were reported, 12 ROI studies and 3 CBR studies. Returns on investment were variable. The median ROI was 2.2 (range 0.7^12^ to 6.2^13^). The median CBR was much higher at 14.4 (range 2^10^ to 29.4^14^).

#### Legislative interventions

Four studies reported on legislative interventions, with substantial returns. The median ROI was 46.5 (range 38^15^ to 55^15^). The median CBR was 5.8 (range 3^10^ to 8.6^16^).

#### Healthcare public health interventions

Six studies reported ROIs in relation to healthcare public health interventions. The ROI median was 5.1, and ranged from 1.15^17^ to 19.35.^18^ No studies reported a CBR.

#### Wider determinants interventions

Twelve studies reported a return on wider determinants interventions (for instance, targeted at children or juvenile offenders). The median ROI was 5.6 (range 1.1^19^ to 10.8^20^) with a median CBR of 7.1 (range 0.66^7^ to 23.6^21^).

#### ROI of public health interventions by level

A total of 29 studies reported returns on investment or CBRs in relation to local public health interventions. The median ROI was 4.1, and ranged from −0.9^22^ to 19.3.^18^ The median CBR was 10.3 (range −0.7^7^ to 23.6^19^).

There were 28 studies reporting on national public health interventions. The median ROI was 27.2 and ranged from −21.3^7^ to 221.^8^ The median CBR was 17.5 (range 1.2^11^ to 167^8^).

## Discussion

### Our systematic review offers several potentially important observations

First, even with the most rudimentary economic evaluations, it was clear that most public health interventions are substantially cost saving. This confirms our theory that public health interventions generally offer a considerable ROI. Median ROI was generally higher than median CBR in all of our key public health expenditure categories. This was because most studies only report one of these two measures, and studies that report ROI tend to have higher estimates. A direct comparison is possible, by converting between ROI and CBR at the study level using the simple formula ROI=CBR**−**1.

Second, we demonstrated a public health ‘effectiveness hierarchy’. Public health interventions at a local level averaged an impressive ROI of 4, meaning that every pound invested yields a return of £4 plus the original investment back. However, ‘upstream’ interventions delivered on a national scale generally achieve even greater returns on investment, particularly legislation (a 10-fold higher ROI averaging 46).

Third, Benjamin Franklin's belief that “an ounce of prevention is worth a pound of cure” is thus borne out by the costs-savings demonstrated, particularly when compared with recent returns for investment in healthcare.^23^ It has been estimated that investing an additional £13 000 in the English National Health Service (NHS) can achieve health benefits of one additional quality-adjusted life year (QALY).^23^ When this health benefit is valued in monetary terms at the UK Department of Health's current rate of £60 000 per QALY,^24^ this represents a ROI of 3.16 (£60 000**−**£13 000/£13 000).

Fourth, this systematic review was partly prompted by recent government cuts to public health budgets in England. We therefore focused on public health interventions delivered in other high-income countries in order to maximise UK relevance. We can therefore now better estimate the likely opportunity costs of the proposed cuts in local and national public health budgets. The median ROI for all public health interventions was 14.3, and the median CBR was 8.3. An ROI of 14.3 implying a cash return of 1430% would sound too good to be true in the financial world. However, public health is different, because decision-making is governed by politics rather than markets. Our systematic review clearly demonstrates that there are big public health investment opportunities out there—they just need some political will to implement them. If we take the lower, conservative CBR figure of 8.3, this would suggest that the opportunity cost of the recent £200 million cuts to public health funding in England is likely to be eightfold higher, in the region of £1.6 billion. The UK government's ‘efficiency savings’ thus represent a false economy which will generate many billions of additional future costs to the ailing NHS and wider UK economy. The recent UK increases in (avoidable) teen pregnancies, sexually transmitted infections, homelessness and suicides are thus predictable and worrying. Do they represent harbingers of worse to come? Although this study draws on the experience of the UK public health system, there are implications for public health systems outside of the UK, which may be guided towards areas of potential underinvestment, and avoid harmful cuts in public health budgets.

### Previous reviews

Ours may be the first comprehensive systematic review of ROI and CBR to include the broad spectrum of public health interventions. Furthermore, it extends and strengthens earlier, narrower reviews which consistently highlighted the cost-effectiveness of selected public health interventions. These included the Australian Assessing Cost Effectiveness (ACE) Prevention Study^25^ and the Health England Leading Priorities (HELP) Tool, which ranked several public health interventions against a set of criteria.^26^ NICE appraises and recommends public health programmes and interventions in England. In 2012, they reviewed 200 cost-effectiveness estimates used in their guidance. Many interventions (particularly around smoking cessation) produced a net cost-saving for the NHS, that is, the intervention was more effective and cheaper than the comparator.^27^ Most interventions were highly cost-effective with a very low cost per QALY: 85% were cost-effective at a threshold of £20 000 per QALY, and 89% at the higher £30 000 threshold, 5% exceeded £30 000 per QALY and only the final 5% were dominated (ie, more costly and less effective than the comparator).^27^

### Health protection interventions

Eighteen studies reported ROI or CBR figures in relation to health protection interventions.^7^ ^8^ ^10^ ^11^ ^28–40^The median ROI for health protection interventions was very high at 34.2. The Australian single measles vaccination programme in the 1980s and 1990s reported the highest CBR, with a CBR of 167:1.^10^ The UK now uses the combined Measles, Mumps and Rubella vaccination that has an excellent ROI of 14:1.^28^

Seven studies assessed the prevention, notification, follow-up and treatment of infectious diseases such as hepatitis B and HIV. Overall, they demonstrated a consistently high ROI, reflecting the high disease burden of infectious diseases and the huge benefits of prevention.^29^

Calculating the ROI of influenza vaccination of healthy working adults is challenging, as it is highly sensitive to the efficacy of the seasonal vaccine. Thus, most such studies^7^ ^30^ have reported a modest twofold ROI overall, but with extreme ROI values ranging from −21 to +174.^30^

### Legislative interventions

One paper reported ROI in relation to legislative interventions, which offered substantial returns on investment, with a median ROI of 46.5. Furthermore, they are relatively low cost and target behaviour at a national level. Introducing a sugar sweetened beverage tax could save $55 for every single dollar invested^15^ in the USA.

### Health promotion interventions

The 15 studies analysing health promotion interventions reported an overall twofold ROI with a more impressive median CBR of 14.4.^10^ ^13^ ^14^ ^22^ ^41–51^ Interventions aimed at reducing rates of falls are able to show one of the swiftest returns on investment of any of the public health interventions identified within this study, with a CBR of 20.6 returned within 18 months.^41^ Falls prevention interventions by their nature are relatively low cost (structured exercise programmes for those at risk of falls), and yet their potential impact on demand management for hospital services is clearly demonstrated. Shifting investment from secondary care for the treatment of falls to primary prevention would show significant and swift returns on investment.

Tobacco control interventions^10^ ^42^ ^43^ overall reported a twofold ROIs, which increased when targeted at high-risk clients such as pregnant women.^42^ Such contrasting results perhaps highlight the complexity of public health interventions.

### Healthcare public health interventions

Six studies^17^ ^18^ ^52–55^ reported healthcare public health results, offering a substantial median ROI of 5.14. The majority focused on disease management or medication adherence for high-risk patients, such as home blood pressure monitoring for hypertension diagnosis and treatment.^18^

### Wider determinants interventions

Twelve studies reported results for wider determinants interventions.^9^ ^10^ ^12^ ^19–21^ ^56–61^ Public health interventions addressing wider determinants also averaged a fivefold ROI. Several studies assessed effectiveness of early years interventions, particularly those targeted at juvenile offenders, or those deemed to be at risk of future offending. Although much of this literature is from the USA^20^ ^21^ ^56^ ^57^ emerging UK evidence demonstrates similar returns to society and the wider economy.^58^ The benefits of early years interventions thus extend far beyond health, with participants reporting improvements in literacy, job prospects and earnings (hence savings to the criminal justice system, increased taxation of higher earnings, etc).

This also highlights the ‘cross-sector flow problem’: cost-effective public health programmes may not be commissioned if decision-makers are only looking through a narrow health lens.

## Strengths

We describe a carefully conducted systematic review. Although the precision of application of ROI calculations varies widely, even the most rudimentary analyses consistently suggest that most public health interventions are substantially cost-saving.

## Limitations

Several limitations should be considered. First, the difficulty of defining what constitutes a ‘public health intervention’, particularly those focused on wider determinants. We purposefully cast the net wide to achieve a broad systematic review. Further analysis of particular topic areas might now be beneficial.

Second, publication bias appears likely, and even some published studies may have been missed. Such studies are inevitably scattered across a wide field of journals and some economic studies may only be available via organisational websites. However, we did search the grey literature and we did identify almost 3000 total studies—a reassuringly high number.

Third, we did not conduct a formal meta-analysis because of the very inconsistent manner in which ROI was calculated, with differing cost perspectives, time horizons and discount rates. Discount rates ranged from 0% to 10%. A high discount rate disadvantages public health interventions that have a long payback time.^62^ Conversely, a 1 year time horizon may offer too short a time frame.

Fourth, the generalisability of the interventions conducted from one country to the next will vary. Participants in US studies may poorly reflect UK demographics and vice versa. Furthermore, some studies focused on vaccination practices that are no longer employed in a number of countries (eg, single measles and haemophilus influenzae type b vaccinations). Similarly, the majority of workplace health promotion initiatives come from the USA, where employers who pay for employees' healthcare will have an additional financial incentive to promote the health of their workforce.

Fifth, the quality of the economic evaluations varied considerably. Practice has clearly improved substantially since the 1970s, with recent evaluations employing more sophisticated modelling techniques. Designing such studies can be challenging as public health interventions are often complex and multifactorial, and it can be difficult to isolate an effect size even within a randomised controlled trial. Some of the published literature may therefore systematically overestimate or underestimate the ROI of interventions, and hence the need for more research.

### Unanswered questions and future research

There is a clear need for further high-quality economic evaluations of public health interventions, which include a range of discount rates and robust sensitivity analyses.

### Implications for clinicians and policymakers

Overall, the results of our systematic review clearly demonstrate that public health interventions are cost-saving, both to health services as well as the wider economy. Furthermore, some are very rapid: falls prevention interventions reported substantial returns within 6–12 months.^41^ One might reasonably expect equally rapid returns for preventive interventions such as immunisation, healthcare, smoking cessation and nutrition.^63^ Although attempting to quantify returns within a short timescale can be challenging, even larger returns on investment were seen over a 10–20 years time horizon.^10^ ^15^ ^17^ ^32^ ^58^ This has significant implications for policymakers, who often work to a much shorter time horizon (typically 3–5 years). We suggest that Public Health England, NICE and other advisory bodies therefore need to routinely emphasise that public health interventions can offer surprisingly rapid returns, which may increase further over the longer term.

## Conclusions

This systematic review suggests that local public health interventions are cost-saving, and offer substantial returns on investment, nationwide programmes even more so.

The cuts to public health budgets therefore represent a false economy. They are likely to generate billions of pounds of additional costs to the health services and wider economy.

**What is already known on this subject**

- It is well known that it is financially preferable for healthcare systems to aim to prevent ill health rather than to subsequently treat it. A number of studies have calculated the return on investment for individual prevention interventions; however, no systematic review has spanned the breadth of public health.

**What this study adds**

- This systematic review demonstrates a median return on investment of public health interventions of ∼14:1. Thus, for every £1 invested in public health, £14 will subsequently be returned to the wider health and social care economy. Furthermore, this review categorises the return on investment according to the public health specialty and local versus national levels of intervention. It suggests that cuts to public health services are short sighted and represent a false economy, with substantial opportunity costs.
