# Supplementary figures and images for: Return on investment of public health interventions: a systematic review
Source: J Epidemiol Community Health. 2017 Mar 29;71(8):827–34. doi: 10.1136/jech-2016-208141 (PMC5537512; doi:10.1136/jech-2016-208141)

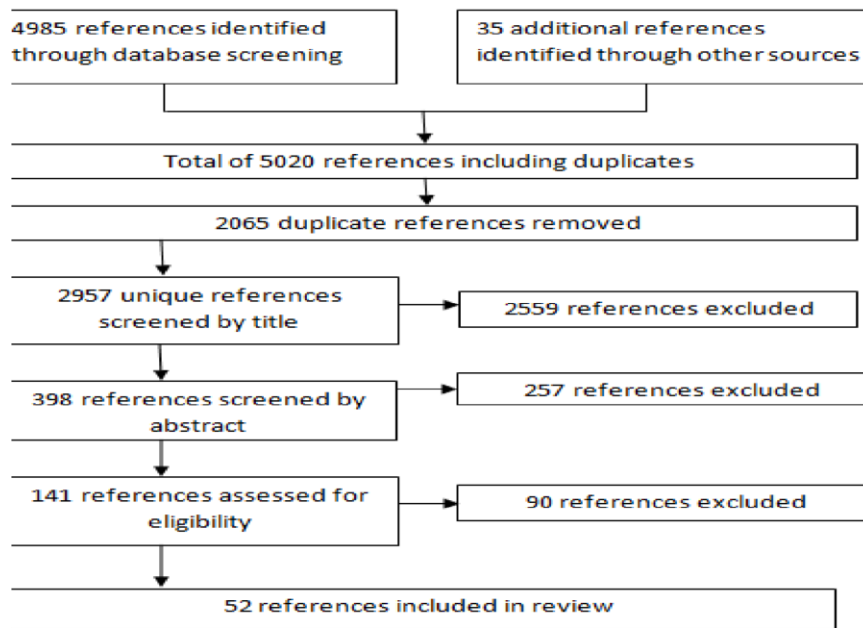

Supplement: supplementary data [file jech-2016-208141supp001.pdf]
